# Editorial: Affective computing and mental workload assessment to enhance human-machine interaction

**DOI:** 10.3389/fnrgo.2024.1412744

**Published:** 2024-05-29

**Authors:** Sabrina Iarlori, Andrea Monteriú, David Perpetuini, Chiara Filippini, Daniela Cardone

**Affiliations:** ^1^Department of Information Engineering, Universitá Politecnica delle Marche, Ancona, Italy; ^2^Department of Engineering and Geology, University “G. d'Annunzio” of Chieti-Pescara, Pescara, Italy; ^3^Lega F. D'Oro Research Center, Osimo, Italy

**Keywords:** human machine interaction (HMI), cognitive and affective states, mental workload (MWL), affective computing, artificial intelligence

In the last years, the use of interactive systems has considerably increased due to a growing interest in human-machine interaction (HMI). The computer technology development and its integration into robot platform have facilitated the HMI, while performing an interactive task. Human Computer Interaction (HCI), described as a discipline concerned with the design, evaluation and implementation of interactive computing systems for human use, is the basis for the Human-Robot Interaction (HRI) (Yanco and Drury, 2002). Overall, the incorporation of human cognitive and affective state monitoring in interactive systems can lead to more efficient and personalized user experiences to control the robot or machine during a real-time interaction. Specifically, the information about the mental workload (MWL), particularly in challenging situations, allows for the ability to automatically adjust the difficulty level of tasks or provide additional support with respect to a recorded mental state change. Moreover, affective computing techniques hold great potential for revolutionizing the way people interact with technology and enhancing the overall quality of user experiences. Considered the technological improvement of mobile and wearable sensors (e.g., wearable and portable electroencephalographic (EEG), functional near infrared spectroscopy (fNIRS) systems and smart devices for physiological signals collection), and contactless devices (e.g., compact infrared thermal and RGB-D cameras), it is possible to better understand the mental user condition and respond to the evaluated cognitive states.

With respect to EEG frequency bands, it is possible to map mental states with attentional, affective and reward-related components elicited by visual stimuli, considering an high variability within and between the involved subjects (Welter and Lotte). By applying artificial intelligence and machine learning algorithms to physiological signals, the authors found that certain EEG patterns were associated with different levels of cognitive load, highlighting the potential of using EEG signals to assess users' mental workload in real-time. Specifically, neuroadaptive systems can be enabled to control the machine's behavior according to the current cognitive and affective users' states. Additionally, Brain Computer Interface (BCI), with feedback regarding the user's emotional state, estimated through EEG, may provide an advantageous framework for affective HCI and emotional cognitive regulation. In this context, the Research Topic aims to collect recent advances in monitoring cognitive and affective states, emphasizing the technological improvements that enhance the HMI in a user-oriented way. The Research Topic, which ended in December 2023, is a collection of four articles written by 16 international authors, and it presents an overview of MWL assessment in HMI scenario, mainly using EEG data analysis.

In this context, Feder et al. have captured the users' brain activity associated with motor responses to different sensory events, extracting event-related potentials (ERPs) from the continuous EEG recording and reflecting the brain's response to a particular event. In this study, participants had to react to auditory events, presented by two different paradigms, in a virtual-reality (VR) setting: by entering codes on virtual keypads to open doors and perceiving sound stimuli externally generated. The study identified a modulation of ERPs' amplitude related to different paradigms. Specifically, the results assessed a relationship between the ERPs' amplitude and the system latency, that should be close to zero to mimic the real world physical interaction. The observed reduction in ERPs' intensity when the events were externally generated indicates a potential difference in cognitive processing when participants are actively engaged compared to passive perception of external stimuli.

Similarly, Gallegos Ayala et al. proposed a BCI approach to provide a real-time and continuous MWL assessment in different scenarios, usable to optimize HCI. In this work, a novel classification algorithm was presented to extract frontal theta oscillations from EEG recordings and to detect MWL during different cognitive tasks. In detail, a published data set that investigated subject dependent task transfer was analyzed through Filter Bank Common Spatial Patterns. The proposed approach enabled a binary classification of MWL with performances of 92.00% and 92.35%, respectively, for either low or high workload vs. an initial no MWL condition.

Vukelić et al. combined a BCI with deep reinforcement learning (RL) for robot training in a 3-D physically realistic simulation environment. The authors applied this method to EEG signals acquired through both wet- and dry-based electrode systems for automatic classification of perceived errors during a robot task. The obtained results indicated that the employment of BCI-based deep RL in combination with the dry EEG-system can significantly accelerate the learning process and offers promising opportunities for training robots and improving their performance in complex tasks.

Welter and Lotte presented an original study where the perception of visual artifacts coincided with aesthetic experiences (AE) that could positively affect health and wellbeing, paving the way for the development of innovative passive BCI systems for enhancing art appreciation. Scientific understanding of the neural dynamics behind AEs, composed of complex cognitive and affective mental and physiological states, would allow a personalized art presentation to improve AE without the necessity of explicit user feedback. The authors proposed a literature review regarding the relationship between EEG rhythm modulation and the attentional, affective, and reward components of AE. This research has important implications for the fields of neuroaesthetics, cognitive neuroscience, and human-computer interaction, offering new possibilities for using technology to improve health and wellbeing through art. The article summarized the state-of-the-art in oscillatory EEG based visual neuroaesthetics and painted a road map toward the development of ecologically valid neuroaesthetic passive BCI systems that could optimize AEs, as well as their beneficial consequences. Authors showed that oscillatory EEG features can contain information about aesthetic preferences and can be highlighted by machine learning approaches.

The works collected by this Research Topic describe a wide range of methods, mainly based on EEG signals. Further studies are indeed necessary to improve the actual knowledge of physiological mechanisms associated with the neurophysiological condition of the user. Moreover, technological enhancements of sensing systems could be crucial for the development of more effective HMI frameworks. In view of the strong interest in the academic and industrial fields, we believe that the present Research Topic will increase rigor and reproducibility in mental workload evaluation research.

## Author contributions

SI: Writing – original draft, Writing – review & editing. AM: Writing – original draft, Writing – review & editing. DP: Writing – original draft, Writing – review & editing. CF: Writing – original draft, Writing – review & editing. DC: Writing – original draft, Writing – review & editing.
